# miR-21, miR-29a, and miR-106b: serum and tissue biomarkers with diagnostic potential in metastatic testicular cancer

**DOI:** 10.1038/s41598-024-70552-x

**Published:** 2024-08-30

**Authors:** Zsuzsanna Ujfaludi, Fruzsina Fazekas, Krisztina Biró, Orsolya Oláh-Németh, Istvan Buzogany, Farkas Sükösd, Tamás Beöthe, Tibor Pankotai

**Affiliations:** 1 Department of Pathology, Albert Szent-Györgyi Medical School, University of Szeged, Szeged, Hungary; 2 Competence Centre of the Life Sciences Cluster of the Centre of Excellence for Interdisciplinary Research, Development and Innovation, University of Szeged, Szeged, Hungary; 3 Department of Urology, Péterfy Sándor street Hospital and Clinic, Budapest, Hungary; 4 Department of Genitourinary Oncology and Clinical Pharmacology, National Institute of Oncology, Budapest, Hungary; 5 Hungarian Centre of Excellence for Molecular Medicine (HCEMM), Genome Integrity and DNA Repair Core Group, University of Szeged, Szeged, Hungary

**Keywords:** MiRNA, Testicular cancer, Cancer diagnostics, Biomarker, MiR-21, MiR-29a, MiR-106b, Cancer prevention, Testicular cancer, Biological techniques, Cancer, Cell biology, Molecular biology, Biomarkers

## Abstract

The imperative need for sensitive and precise tools is underscored in cancer diagnostics, with biomarkers playing a pivotal role in facilitating early detection and tumor diagnosis. Despite their classical pathological classification, testicular tumors lack valuable markers, emphasizing the necessity to identify and apply serum tumor markers in clinical management. Unfortunately, existing biomarkers exhibit limited sensitivities and specificities. Recent years have witnessed the discovery of novel RNA molecules, presenting a potential breakthrough as diagnostic tools and promising biomarkers. This report presents compelling evidence supporting the detection of early testicular cancer by applying a set of nine microRNAs (miRNAs), establishing them as valuable serum biomarkers for diagnosis. We developed a standardized serum-based measurement protocol and conducted comprehensive statistical analyses on the dataset to underscore the diagnostic accuracy of the miRNA pool. Notably, with a sensitivity exceeding 93%, miR-21, miR-29a, and miR-106b surpass classical serum tumor markers in the context of testicular cancer. Specifically, these miRNAs are poised to enhance clinical decision-making in testicular cancer detection and hold the potential for assessing tumor growth in monitoring chemotherapy outcomes.

## Introduction

Testicular cancer (TCa) is a rare disease that accounts for 1–2% of all malignant tumors in male patients^1^. However, it represents the most common solid tumor among young men (aged 15–40)^2^, with a continuous increase in incidence during recent decades^1,2^. According to the Globocan 2020 database, about 74,400 new cases of TCa were diagnosed, and over 9300 TCa-related deaths occurred in 2020 worldwide^3^. The predominant histology (95%) of neoplasms in the testis are testicular germ cell tumors (TGCT)^4^. Post-pubertal TGCT has few DNA mutations and somatic changes; however, a restricted region of 12p is overexpressed in most cases^5^. Seminomas and bilateral TGCTs frequently present cKIT mutations^6^. These mutations are necessary for the non-invasive precancerous cells to progress into invasive TGCT^7^. Patients with TGCT are also four times more likely to carry germline loss-of-function checkpoint kinase 2 (*CHEK2*) variants than those not diagnosed with TGCT^8^. Gain of chromosomes X, 7, 8, and 21 and loss of chromosomes Y, 1p, 11, 13, and 18 are also characteristic of post-pubertal TGCT^9^. Due to landmark treatment advances introduced in the late 1970s, TGCT became a highly curable disease, even in advanced, metastatic stages^10^. The 5-year survival rate is 95%, primarily due to platinum-based combination chemotherapy and advanced salvage treatment options^11^. Thus, treatment at high-volume specialist centers, contributing to significantly higher overall survival and lower relapse rates, is highly recommended^12,13^. Clinical management of TGCT is guided by classic serum tumor markers (lactate dehydrogenase [LDH], alpha-fetoprotein [AFP], and beta-human chorionic gonadotropin [β-hCG]) and imaging diagnostics^14^. These historically used markers, however, are only elevated in 60% of the cases at diagnosis^15^, have limited specificity,^16^ and show no correlation with the post-chemotherapy residual tumor histology^17^.

Salvage retroperitoneal lymph node (LN) dissection (RPLND) is recommended in cases of incomplete response to systemic chemotherapy with strong evidence^18^. Surgical resection of post-chemotherapy residual masses which are > 1 cm in size is mandatory according to current urooncology guidelines^19,20^, except in cases of pure seminoma. 40–50% of resected masses harbor only necrotic or fibrotic tissue with no evidence of viable tumor or teratoma^20^. A viable tumor is found in 6–10% of cases^20^ with a relatively poor prognosis^21^. While teratomas usually behave indolently, they can undergo malignant transformation or grow in size, causing the compression of vital organs^22,23^; therefore, their surgical removal is necessary. If the presence of a viable tumor or a teratoma in the residual lesion could safely be ruled out, surgical interventions with possible complications should be avoided.

Developing more reliable tumor markers could be an invaluable tool for TGCT management, from diagnosis through early staging to monitoring treatment response and the assessment of post-chemotherapy residual lesions. The novel markers could shape follow-up practices and reduce the burden of CT imaging. Liquid biopsy represents an evolving frontier in diagnostics, harnessing nucleic acids from biofluids such as DNA and RNA^24^. The analysis of circulating tumor DNA (ctDNA) stands out, offering crucial insights into genetic alterations and tumor dynamics, thereby advancing the management of diverse diseases. Beyond ctDNA, recent investigations have underscored the substantial potential of micro-RNA (miRNA) classification from blood sera in identifying cancer^25,26^. These ~ 22 nucleotides long single-stranded circulating non-coding miRNAs, packed in exosomes, apoptotic bodies, or associated with Argonaute proteins, boast stability and can be readily detected with minimally invasive procedures (e.g., blood sampling)^27^. Hence, they emerge as prospective biomarkers for tumor prognosis, early diagnosis, and potential therapeutic interventions. MiRNAs bind target mRNAs and regulate post-transcriptional gene expression through mRNA silencing^28^. MiRNAs are frequently dysregulated in tumors, acting as either tumor suppressors or oncogenes^29–31^. Despite the ongoing expansion of our understanding regarding their functions across various tumor types, the full exploration of their application as biomarkers in cancer prognosis, diagnosis, or therapy remains a subject of ongoing investigation.

Numerous circulating miRNA biomarkers have recently been explored for various cancers; however, their applicability has not yet met diagnostic standards. To address this issue, we conducted a comprehensive database search which identified nine miRNA candidates. Subsequently, we performed qPCR measurements on TCa patients' sera samples, revealing a significant downregulation of miR-21, miR-29a, and miR-106b levels in individuals with metastatic TCa. Based on these findings, we propose that these three miRNAs hold potential biomarkers in the diagnostic context of TCa.

## Materials and methods

### Sample collection, cohort criteria, and clinical data

All healthy individuals were considered normal (N) control donors not diagnosed with tumorous maladies. The patients involved in the study were previously diagnosed with TCa (T). All of them underwent chemotherapy treatment and showed incomplete responses in radiological examinations. Residual metastatic LNs located in the abdominal cavity were removed during RPLND surgery, followed by histological assessment by expert uropathologists. Blood tests were conducted on TCa patients one day before the RPLND surgery.

Blood samples were taken in separator gel containing test tubes and then chilled on ice for 30 min. Sera samples were separated with centrifugation (2000 rpm, 10 min, 4 °C), aliquoted in nuclease-free cryotubes, and snap-frozen in liquid nitrogen. Tissue samples were collected from TCa patients upon salvage RPLND (n = 18), second look RPLND (n = 3), and radical inguinal orchiectomy (n = 1). Immediately after surgical removal, representative areas of the removed tumors were resected, cut into pieces (for parallel histological examination and miRNA testing), and snap-frozen in liquid nitrogen. All the collected sera and LN samples were kept at − 80 °C for a maximum of 1 year and a more extended period, respectively. All samples were anonymized upon blood taking and RPLNDs.

All clinical data, including age, body mass index (BMI), lifestyle habits, other chronic diseases, and familial involvement in cancer, were collected on sheet forms voluntarily filled out by the donors. Primary diagnosis of TCa and the histology of LNs removed during RPLND surgery were recorded by the actual healthcare institutes.

All donors signed written informed consent forms. The study was conducted with the approval of the Scientific and Research Ethical Committee of the Hungarian Scientific Council (IV-5376-2 TUKEB ETT), and the experiments conform to the Declaration of Helsinki in 1995 (revised in Edinburgh in 2000).

### Serum miRNA purification, reverse transcription, and quantitative RT-PCR

Hemolysis of sera was measured with NanoDrop 2000 spectrophotometer (Thermo Fisher Scientific, USA). Serum miRNAs were purified using ReliaPrep miRNA Cell and Tissue Miniprep System (Promega, USA) according to the manufacturer's recommendation. All tissue samples were embedded in Cryomatrix (ThermoFisher Scientific, USA) and sliced into 10 µm sections with CryoStar NX50 microtome (ThermoFisher Scientific, USA). The opening and closing sections were hematoxylin–eosin stained^32^ and assessed by expert uropathologists. Five sections of each LN sample were used for miRNA extraction using ReliaPrep miRNA Cell and Tissue Miniprep System (Promega, USA) according to the manufacturer's protocol. Equal volumes of extracted miRNA samples were reverse transcribed with MystiCq® microRNA cDNA Synthesis Kit (Sigma-Aldrich, USA). Oligos for miRNA-specific quantitative reverse transcription PCR (qRT-PCR) amplification were designed with miRprimer_2 software^33^. Primer sequences are represented in Table 1. The qPCR reactions were performed with the Rotor-Gene Q 5plex HRM Platform (Qiagen, Germany) using SYBR Green chemistry (GoTaq® qPCR Master Mix, Promega, USA). Normalized serum levels of miRNAs were calculated with the –∆Ct method using the Cq values of U6 as endogenous control and are indicated as log_2_ fold-change in the figures.

**Table 1:** Sequences of miRNA-specific primers used in qRT-PCR reactions.

miRNA	Sequence (5′–3′)	Accession nr	miR-sequence
hsa-miR-19a	GCAGTGTGCAAATCTATGC	MIMAT0000073	UGUGCAAAUCUAUGCAAAACUGA
hsa-miR-21	TAGCTTATCAGACTGATGTTGA	MIMAT0000076	UAGCUUAUCAGACUGAUGUUGA
hsa-miR-29a	TAGCACCATCTGAAATCGG	MIMAT0000086	UAGCACCAUCUGAAAUCGGUUA
hsa-miR-106b	TAAAGTGCTGACAGTGCAG	MIMAT0000680	UAAAGUGCUGACAGUGCAGAU
hsa-miR-155	CGCAGTTAATGCTAATCGTGATAG	MIMAT0000646	UUAAUGCUAAUCGUGAUAGGGGUU
hsa-miR-199a	GCCCAGTGTTCAGACTAC	MIMAT0000231	CCCAGUGUUCAGACUACCUGUUC
hsa-miR-367	GCAGAATTGCACTTTAGCAATG	MIMAT0000719	AAUUGCACUUUAGCAAUGGUGA
hsa-miR-371a	GTGCCGCCATCTTTTGAG	MIMAT0000723	AAGUGCCGCCAUCUUUUGAGUGU
hsa-miR-373	GAAGTGCTTCGATTTTGGG	MIMAT0000726	GAAGUGCUUCGAUUUUGGGGUGU
U6	CTCGCTTCGGCAGCACATA	Gene ID: 26,827	

### Biostatistics

Statistical comparisons of the normal and tumorous datasets were performed with SigmaPlot 12.5 software package (Systat Software Inc., USA). The distribution of datasets was examined using the Shapiro–Wilk normality and Equal Variance test. The values of variances were determined with ANOVA or Kruskal–Wallis ANOVA on Ranks methods, followed by multiple comparisons using the Holm–Sidak test or Tukey's test, respectively. Multiple comparisons were carried out with one-way ANOVA, followed by Dunn's- or Dunnett's post-hoc tests, depending on the distribution of the analyzed dataset. Diagnostic abilities and cut-off values were defined with the Receiver Operating Characteristic curve (ROC curve) analysis tool of SigmaPlot 12.5 software using a 99% confidence interval. Variance-based statistics were fulfilled using the ClustVis online toolset (https://biit.cs.ut.ee/clustvis/). For heatmap generation, data were clustered based on Euclidean distance and average linkage methods.

### miRNAs

miR-19a, a member of the miR-17–92 cluster on chromosome 13q31.3, targets phosphatase and tensin homolog deleted on chromosome ten (PTEN), therefore contributing to the activation of the phosphatidylinositol 3'-kinase (PI3K)–Akt surviving promoting pathway^33^. One of the most important oncomir, miR-21, has a regulatory role in many processes, from promoting cell proliferation through DNA repair to the induction of angiogenesis^34–36^. Many studies have described miR-29a as a tumor suppressor; however, it influences diverse oncogenic processes, including regulation of epigenetics, facilitating proliferation, angiogenesis, and immunomodulation^37^. The overexpression of miR-106b, a member of the miR-106b-25 cluster, was associated with aggressive tumor phenotypes, indicating its crucial role in the regulation of multiple signaling pathways involved in tumor progression and also acquired resistance to anti-tumoral therapies^38–40^. Facilitating tumor growth, miR-155 has a regulatory role in inflammation and has been reported as a promising biomarker for monitoring breast cancer in sera^41–43^. Promoter methylation-directed downregulation of miR-199a and the resulting invasive phenotype via the overexpression of podocalyxin-like (PODXL) anti-adhesion transmembrane protein was proved in TCa in various studies^44^. A promising diagnostic value of circulating miR-367, miR-371a, and miR-373 has already been demonstrated in multiple TCa cohort studies. The primer sequences used for qRT-PCRs are represented in Table 1.

## Results

### Cohort characteristics

Twenty-seven healthy male volunteers with a mean age of 42.9 (ranging between 20 and 66) and 27 clinically LN-positive TGCT patients with a mean age of 36.0 (ranging from 20 to 51) were involved in our study cohort. 7.4% of TCa patients (2 out of 27) were in complete regression, having burn-out tumors at the time of primary diagnosis. 18.5% (5 out of 27) of the patients were diagnosed with classical seminoma, and the others carried mono-component or mixed germ cell tumor (GCT), representing 14.8% (4 out of 27) and 55.6% of the T group (15 out of 27), respectively. Post-chemotherapy mature teratoma was confirmed in 3.7% (1 out of 27) of the cases from an orchiectomy specimen.

Blood serum miRNA expression was analyzed in all 27 TCa patients, 25 of whom underwent salvage RPLND, while in 2 cases, only post-chemotherapy orchiectomy was performed at our referral center.

TGCT patients were classified according to their clinical stage (TNM classification) and resected LN histological subtype (viable GCT, teratoma, and necrosis/fibrosis) (Table 2).

**Table 2:** Post-chemotherapy clinical stage and LN histology of the cohort.

Post-chemotherapy clinical stage			
Orchiectomy only	cN1	cN2	cN3
n = 2 (7.4%)	n = 5 (18.5%)	n = 9 (33.3%)	n = 11 (40.7%)

LN histology			
Fibrosis, necrosis	Teratoma	Viable tumor	
n = 10	n = 12	n = 3	

On follow-up imaging after chemotherapy, the residual lesions of RPLND patients were as follows: 18.5% cN1, 33.3% cN2, and 40.7% cN3. 12% of resected LNs harbored viable GCT, 48% teratoma, and complete regression (fibronecrotic tissue) was found in the rest of the tumors (n = 10 [40.0%]). Three (12%) patients developed distal metastases after chemotherapy (Table 2).

All retroperitoneal LNs were resected from the affected surgical template in accordance with the Heidenreich criteria. Pathological LNs were selected for sampling, from which 33 retroperitoneal LNs of 22 patients were included for miRNA expression analysis (metastatic LNs of 5 donors were unavailable).

Further assessing the donors' basic medical parameters, data about their lifestyle—including smoking and alcohol intake—, other diagnosed chronic diseases, and history of familial cancer cases were recorded. Some medical data are not represented in the cohort database, as providing these personal details was voluntary. The average BMI (i.e., physique) was similar in the two groups. More active smokers were found in the T group than in the N category (37.0% and 29.6%, respectively). Those who quit smoking were represented in higher counts among the healthy donors (25.9%) than the T group (11.1%). The proportions of non-smokers were similar in the groups (40.7% and 44.4%). Additional chronic illnesses occurred more frequently in patients with TCa than in healthy individuals (48.1% and 33.3%, respectively). The healthy donors' familial cancer incidence was higher than that of TCa patients (37% and 29.6%, respectively) (Table 3).

**Table 3:** Metadata of donors.

		No. of donors	Age		BMI	
Cohort characteristics						
N group		27	42.9 ± 12.0		26.9 ± 5.4	
T group		27	36.0 ± 9.2		27.85 ± 7.5	
Total no. of donors		54	–		–	
Total no. of LNs		33	–		–	
No. of LN samples/patients		0	1	2	3	4
No. of TCa patients		5	9	11	1	1
Detailed data of N group (%)						
Category/score	n.a	0	1	2	3	4
Smoker	3.7	40.7	25.9	29.6	–	–
Alcohol intake	3.7	55.6	7.4	3.7	14.8	14.8
Additional chronic illness(es)	3.7	63.0	33.3	–	–	–
Familial anamnesis for cancer	11.1	51.9	37.0	–	–	–
Detailed data of T group (%)						
Category/Score	n.a	0	1	2	3	4
Smoker	7.4	44.4	11.1	37.0	–	–
Alcohol intake	7.4	51.9	33.3	0.0	0.0	7.4
Additional chronic illness(es)	7.4	44.4	48.1	–	–	–
Familial anamnesis for cancer	11.1	59.3	29.6	–	–	–
Primary GCT histology	0.0	7.4	18.5	14.8	55.6	3.7
LN histology	0.0	15.2	51.5	33.3	–	–

The abbreviations used in the table stand for the following: N: healthy donors, T: donors diagnosed with testicular cancer (TCa), and LN: lymph node. Scores for the "Smoker'' category are as follows: n.a.: no data available, 0: non-smoker, 1: ex-smoker, and 2: smoker. Scores for the "Alcohol intake" category are as follows: n.a.: no data available, 0: never, 1: occasionally, 2: monthly, 3: weekly, and 4: daily. Scores for the "Additional chronic illness(es)" category are as follows: n.a.: no data available, 0: no other illness(es), and 1: having other chronic illness(es). Scores for the "Familial anamnesis for cancer" category are as follows: n.a.: no data available, 0: none, 1: one or more relatives were diagnosed with any cancer. Scores for the "Primary GCT histology" category are as follows: 0: burned-out tumor, complete regression, 1: Seminoma, 2: Nonseminomatous GCT—monocomponent, 3: Mixed GCT, and 4: Post-chemotherapy teratoma. Scores for the "LN histology" category are as follows: 0: reactive (inflammation), 1: teratoma, and 2: no living tumor.

### Pre-selected sera miRNA expression profiles can differentiate between TCa patients and healthy individuals

**Fig. 1:**
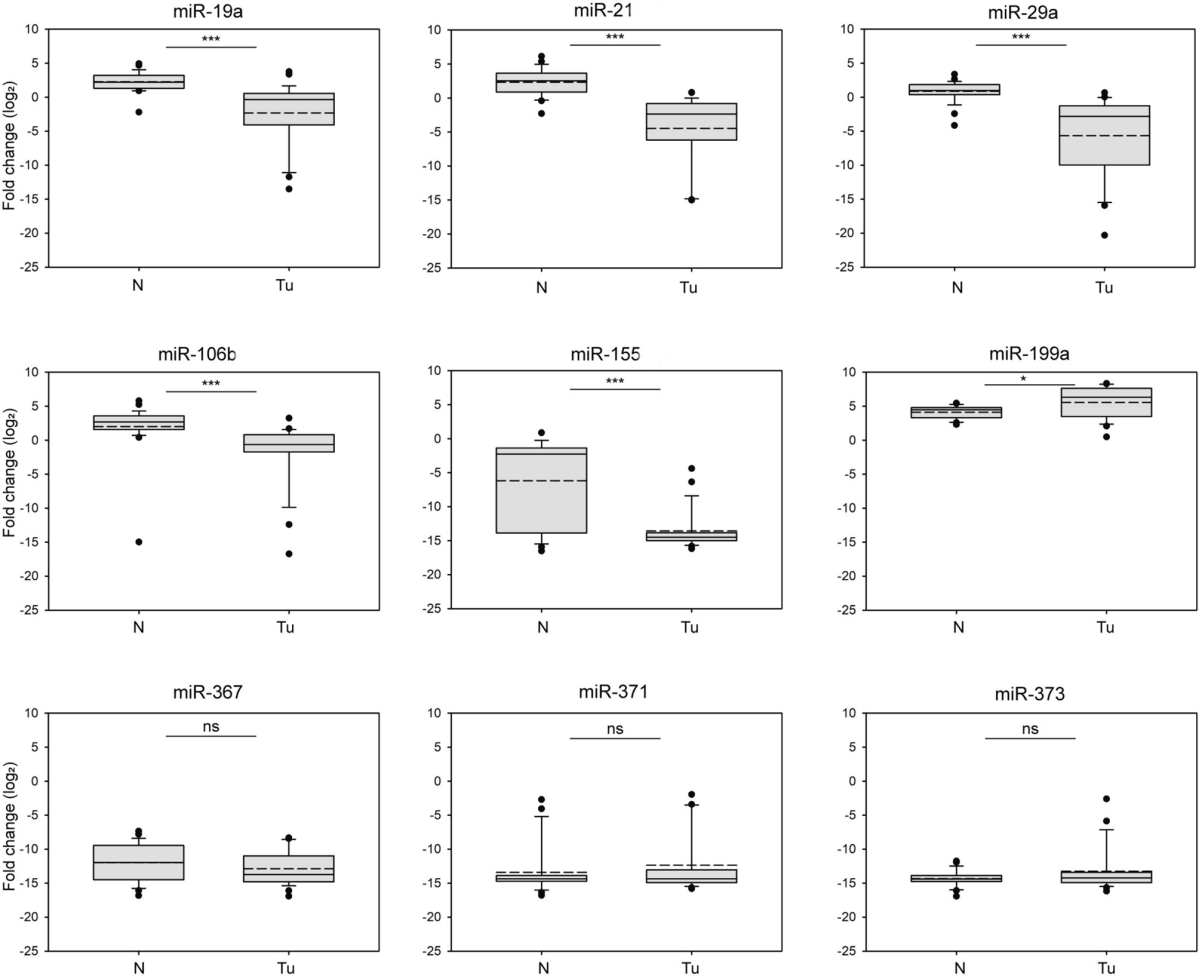
Selected miRNAs display different serum levels in TCa patients than in healthy individuals. The relative amounts of circulating miRNAs in TCa patients were compared to those of healthy donors and statistically evaluated with ANOVA. The normalized levels of circulating miRNAs are displayed on box plots and indicated as log2 fold change. Abbreviations stand for the following: N = normal (healthy donors), Tu: tumor (TCa patients serum sample). Stars indicate significant differences: *P < 0.05, ***P < 0.001, and ns = non-significant. Solid lines represent means, and medium dashed lines indicate the medians of datasets.

A panel of candidate diagnostic miRNAs was established to compare the serum miRNA expression profiles of TCa patients to healthy individuals. Based on literature data, nine miRNAs were selected according to their general oncogenic, tumor-suppressing, or dual role in tumorigenesis, including their specific dysregulation in TCa^34^. After determining the serum expression levels of each candidate miRNA, the differential expressions were calculated and statistically evaluated. Five candidates—miR-19a, miR-21, miR-29a, miR-106b, and miR-155—showed significant levels in the T group compared to the N group. miR-199a was significantly upregulated in TCa patients; however, the difference was much less dramatic than in the previously listed five miRNAs (Fig. 1). In the case of two members of the miR-371–373 cluster, miR-371a and miR-373, the expression pattern was similar between TCa patients and healthy individuals (Fig. 1). However, most of the oncomiRs, such as miR-21, are overexpressed in human tumors^35,36^; interestingly, we found most of them reduced in the sera derived from TCa patients. This seemingly controversial result may reflect the nature of the cohort as TCa patients underwent orchiectomy and chemotherapy; therefore, they did not bear the primary tumor, and the metastatic LNs responded partially to systemic treatment.

**Fig. 2:**
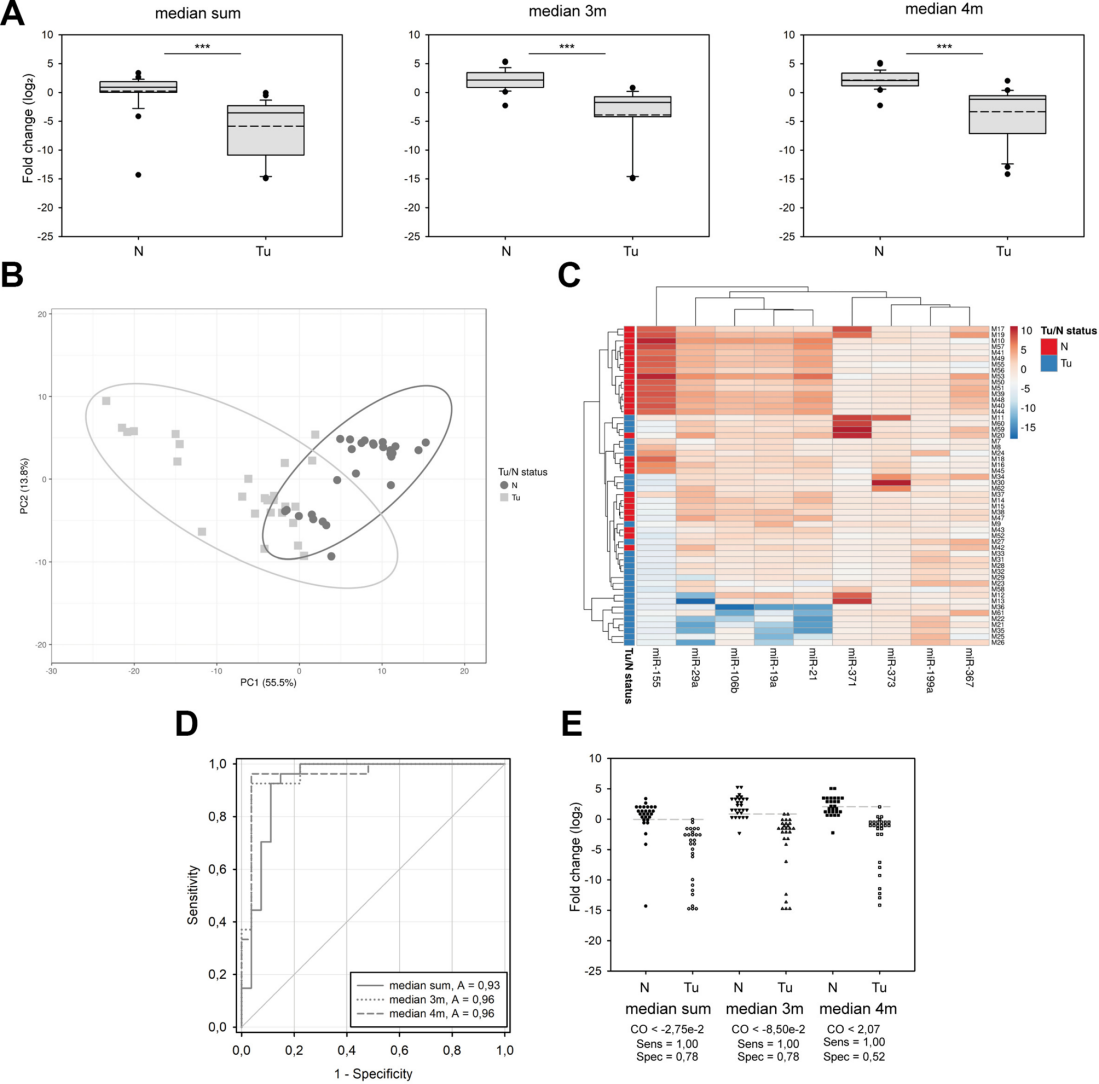
Serum expression patterns of selected oncomiR combinations have a diagnostic ability in TCa post-chemotherapy monitoring. Medians of normalized expressions of all or selected miRNAs were calculated and compared in the two-sample groups and indicated as log2 fold change. (**A**) Left: Box plots of medians of all nine miRNAs (median sum). Middle: Box plots of medians of miR-21, miR-29a, and miR-106b (median 3 m). Right: Box plots of medians of miR-19a, miR-21, miR-29a, and miR-106b (median 4 m). Solid lines represent means, and the medium dashed lines indicate the medians of datasets. (**B**) The principle component analysis (PCA) plot of the 9 miRNA datasets generated with the ClustVis online tool. (**C**) Hierarchical clustering of the nine oncomiRs and the enrolled individuals' samples represented by a heat map and generated with the ClustVis online tool. (**D**) A receiver operating characteristic curve (ROC) analysis of the median all miRNAs, median 3 m, and median 4 m indicators, area under the curves (AUCs) are highlighted. E) Scatter plots of the 9 miRNAs, 3 m, and 4 m in the control and TCa samples. The grey dashed line shows the cut-off value of the tests. Abbreviations stand for the following: N = normal (healthy donors), Tu: tumor (TCa patients serum samples). Stars indicate significant differences: ***P < 0.001, and ns = non-significant, calculated using ANOVA. Solid lines represent means, and medium dashed lines indicate the medians of datasets.

We used the medians of normalized values for each sample to gain a complete serum expression pattern of these pre-selected circulating miRNAs. Including all nine miRNAs (i.e., "median sum") in the calculation, we found a significant difference between the two cohort groups (Fig. 2A); however, the data possessed a relatively high standard deviation in TCa patients (in the case of sera samples, referred to as Tu). Next, we focused only on those miRNAs that showed the highest significant difference between TCa patients and healthy individuals. We established two overlapping groups of miRNAs: (I) "median 3m" included miR-21, miR-29a, and miR-106b and (II) "median 4m" implicated miR-19a, miR-21, miR-29a, and miR-106b. Both indicators displayed significant differences between the control and Tu groups (Fig. 2A). Although miR-155 showed a significant difference between the two examined groups, the expression data of miR-155 showed quite diverse distribution in the control samples, in many cases without any detectable serum levels; thus, it was regarded as a non-reliable factor and disclosed from these analyses with medians (Fig. 1). These results indicate that the serum signatures of circulating miRNAs can distinguish between healthy individuals and TCa patients and presume a possible diagnostic value of these pre-selected miRNAs. To underline this hypothesis, we performed Principal Component Analysis (PCA) based on the normalized serum levels of the nine miRNAs. The N and Tu groups formed discreet, mildly overlapping clusters (PC1 + PC2 components depict 69.3% of the total variance, Fig. 2B). Hierarchical clustering of the miRNAs and samples displayed a similar dissociation (Fig. 2C, Y-axis). The miRNAs formed three distinct clusters: (I) miR-367, miR-371a, and miR-373, (II) miR-19a, miR-21, miR-29a, and miR-106b, and (III) miR-155 (Fig. 2C, X-axis). The median values of the first two clusters were significantly different in TCa patients than in the control group (Fig. 2C). However, miR-155 was distinguished from the other two clusters, having a unique intensity pattern throughout the samples, reflecting the high variance of the detected serum levels of this marker miRNA.

We performed ROC curve analyses to determine the diagnostic ability of our pre-selected marker miRNA panel. The medians of all nine miRNAs (median sum) were able to discriminate between the normal and TCa tumorous sample population with 93% possibility (Area Under the ROC Curve (AUC) 0.93, Fig. 2D). The test's specificity is 78%, with a threshold of 0.0275 (Fig. 2D). The discriminative abilities were even higher (96%) in both cases of the summarised expression patterns of the significantly altered miRNAs (median 3 m and median 4 m vs. median sum, Fig. 2E). However, the specificity of these indicators as diagnostic markers was different as the "median 3 m" showed 78%, whereas the "median 4 m" possessed only 52% (Fig. 2E). These results indicated that using the serum expression patterns of miRNAs—particularly that of miR-21, miR-29a, and miR-106b—may have a diagnostic ability in TCa patients' follow-up after chemotherapy.

### miR-21, miR-155, and miR-373 oncomiRs express differentially in teratoma-infiltrated metastatic lymph nodes

**Fig. 3:**
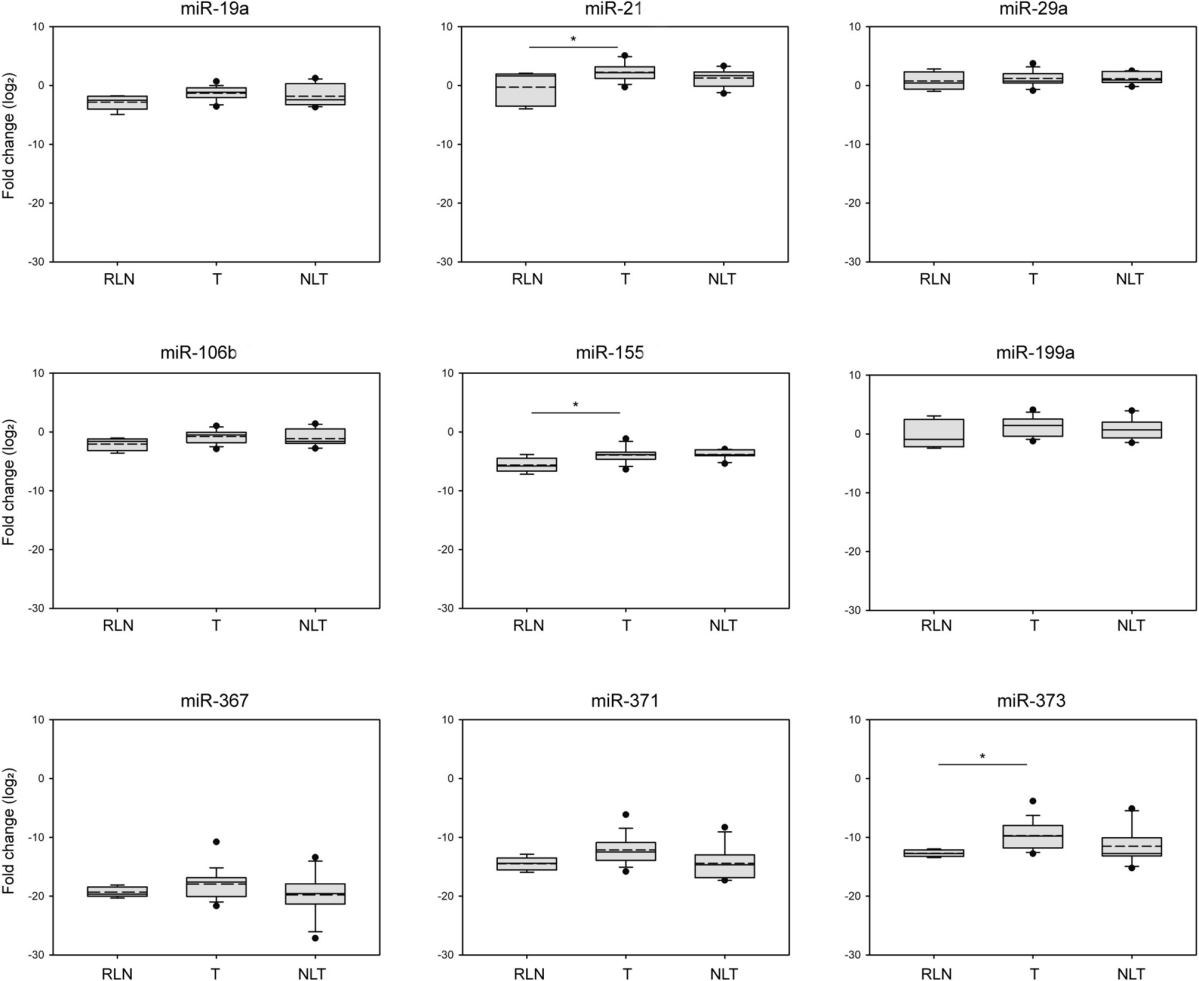
miR-21, miR-155, and miR-373 oncomiRs are expressed differentially in teratoma-infiltrated metastatic lymph nodes. The relative tissue expression levels of miRNAs in LNs derived from TCa patients were compared to those of healthy donors and statistically evaluated with ANOVA. The normalized levels of miRNAs are displayed on box plots and indicated as log2 fold change. Abbreviations stand for the following: RLN = reactive LNs, T = teratoma (metastatic LNs with living teratoma cells), NLT = no living tumor (LNs with scar tissue or necrosis). Stars indicate significant differences, *P < 0.05, calculated using ANOVA. Solid lines represent means, and the medium dashed lines indicate the medians of datasets.

This study aims to develop more reliable tumor markers for TGCT management to monitor treatment responses and assess post-chemotherapy residual lesions. Therefore, we examined the tissue levels of the previously selected miRNAs in LNs removed by salvage RPLND surgeries. To explore whether the expression of any of the marker miRNAs correlates with the statuses of the LNs, we first scored the TCa patients into three groups according to the histological assessment of the removed metastases. Those who possessed inflamed LNs (15.2%) were recruited in the "reactive LN" (RNL) group. In 33.3% of the patients, the removed LNs were necrotic or scar tissue was detected due to chemotherapy, standing for the "no living tumor" (NLT) group. The largest sub-group, representing 51.5% of the involved TCa patients, included the teratoma-containing LNs ("teratoma group", T) (Table 2). None of the nine miRNAs showed significantly altered expression in the NLT samples compared to the other two categories. Mildly significant differences were detected between RLN and T groups in the case of miR-21, miR-155, and miR-373 (Fig. 3).

**Fig. 4:**
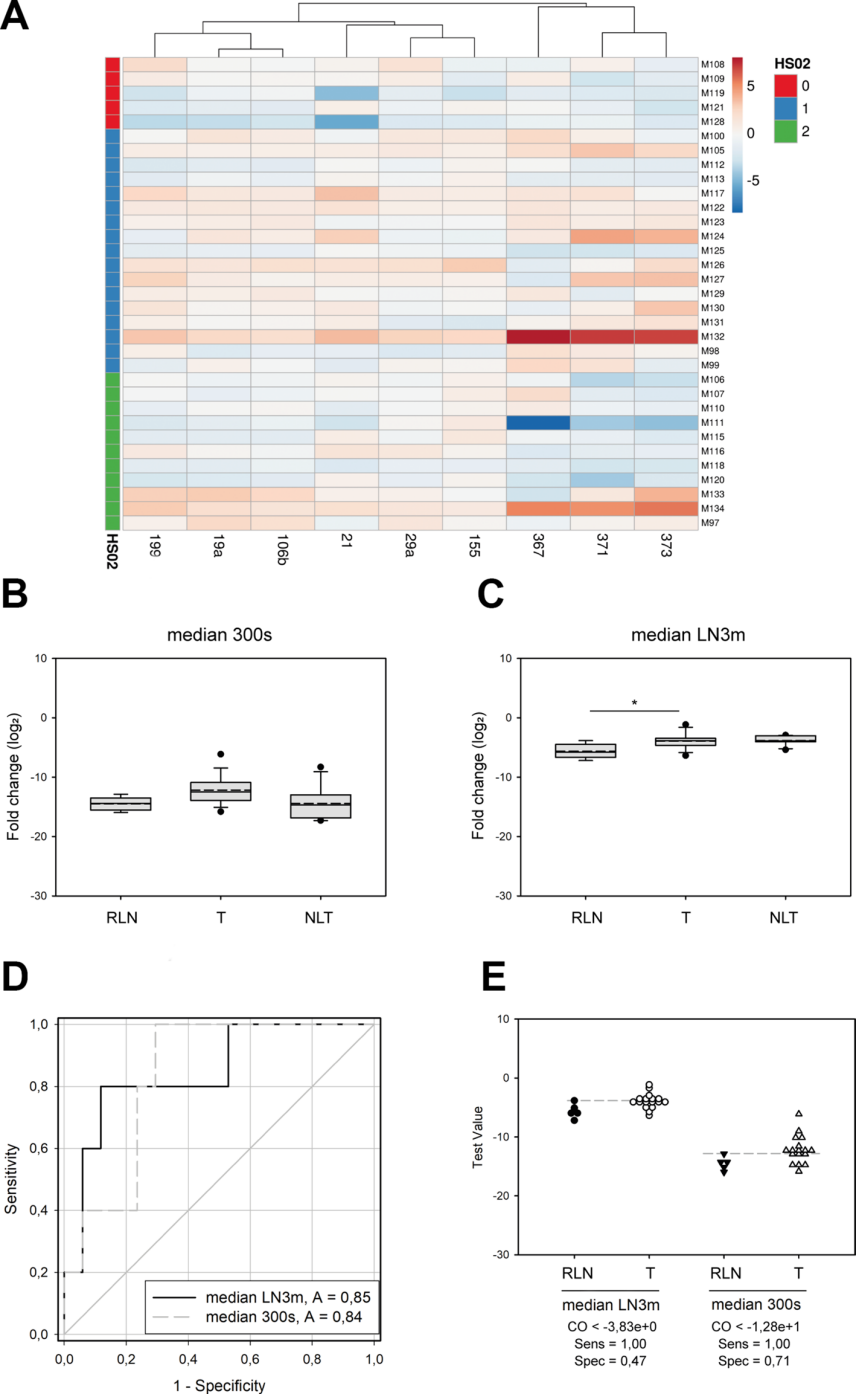
The tissue expression patterns of unique combinations of specific miRNAs differ in inflamed LNs from teratoma-infiltrated metastatic TCa lesions. (**A**) Heat map of the nine oncomiRs in RLNs, teratoma-infiltrated metastatic LNs, and necrotic LNs/scar tissue generated with the ClustVis online tool. Histology scores (HS) are indicated on the Y-axis as 0: RLNs, 1: teratoma, 2: scar tissue or necrotic LNs. (**B**) Box plots of medians of miR-367, miR-371, and miR-373 (median 300 s). (**C**) Box plots of medians of miR-21, miR-29a, and miR-155 (median LN3m). Solid lines represent means, and the medium dashed lines display the medians of datasets. The normalized tissue expression levels of miRNAs are indicated as log2 fold change. (**D**) ROC curve analysis of the median LN3m and median 300 s indicators; AUCs are highlighted. (**E**) Scatter plots of the median LN3m and median 300 s indicators in the control and TCa samples. The grey dashed line shows the cut-off value of the tests. Abbreviations stand for the following: HS = histology score, RLN = reactive LNs, T: teratoma (metastatic LNs with living teratoma cells), NLT: no living tumor (LNs with scar tissue or necrosis), A = AUC value, CO = cut-off value, Sens = sensitivity, and Spec = specificity. Stars indicate significant differences, *P < 0.05, calculated using ANOVA and post-hoc tests.

Using variance-based statistical analyses, the pattern of the nine miRNAs was not specific for any of the three LN categories, as none of the sample groups clustered together in PCA analysis or hierarchical clustering represented with a heat map (Figure S1). Nevertheless, when a similar analysis was performed on the scored sample sets, three groups of the marker miRNAs were separately clustered (Fig. 4A, Y-axis): (I) miR-367, miR-371a, and miR-373, (II) miR-21, miR-29a, and miR-155, and (III) miR-199, miR-19a, and miR-106b. To examine whether the summarised expression patterns of the first two miRNA clusters are sufficient to distinguish between the LN sub-groups, we calculated and then compared the medians of the miR-367 + miR-371a + miR-373 and miR-21 + miR-29a + miR-155 trios separately, referred to as "median 300 s'' and "median LN3m,'' respectively. However, the differences in median values between the three-sample categories were greater than would have been expected by chance (ANOVA, P = 0.021); the pairwise comparisons did not prove any distinctive sub-group-specific characteristics (post-hoc test by Dunn's Method, P > 0.05) (Fig. 4B,C). A similar analysis of the median LN3m indicator revealed a significant diversity of teratoma samples compared to the reactive LNs (post-hoc test by Dunnett's Method, P < 0.05) but not to the NLT group. These data suspect that the tissue expression patterns of specific miRNAs' unique combinations may potentially differentiate inflamed LNs from teratoma-infiltrated metastatic TCa lesions.

We performed ROC analyses to address the possible diagnostic ability of median 300 s and LN3m indicators, including only the RLN and T sub-groups of LNs (Fig. 4D). However, the two indices revealed good testing abilities since the AUC values were 0.84 and 0.85, respectively, but the specificities were relatively weak (71% and 47%, respectively) (Fig. 4D,E). Our data thus showed distinct differences in the tissue expression levels of sets of oncomiRs between reactive and metastatic LNs with living teratoma. Besides, evaluating the possible diagnostic utilities of these indices has limitations using this cohort since retroperitoneal LNs from healthy individuals were not available. Additionally, the sample numbers of the scored sub-groups were unequal and relatively low.

### The serum levels of circulating miRNAs show no differences between the distinct chemotherapy responders

**Fig. 5:**
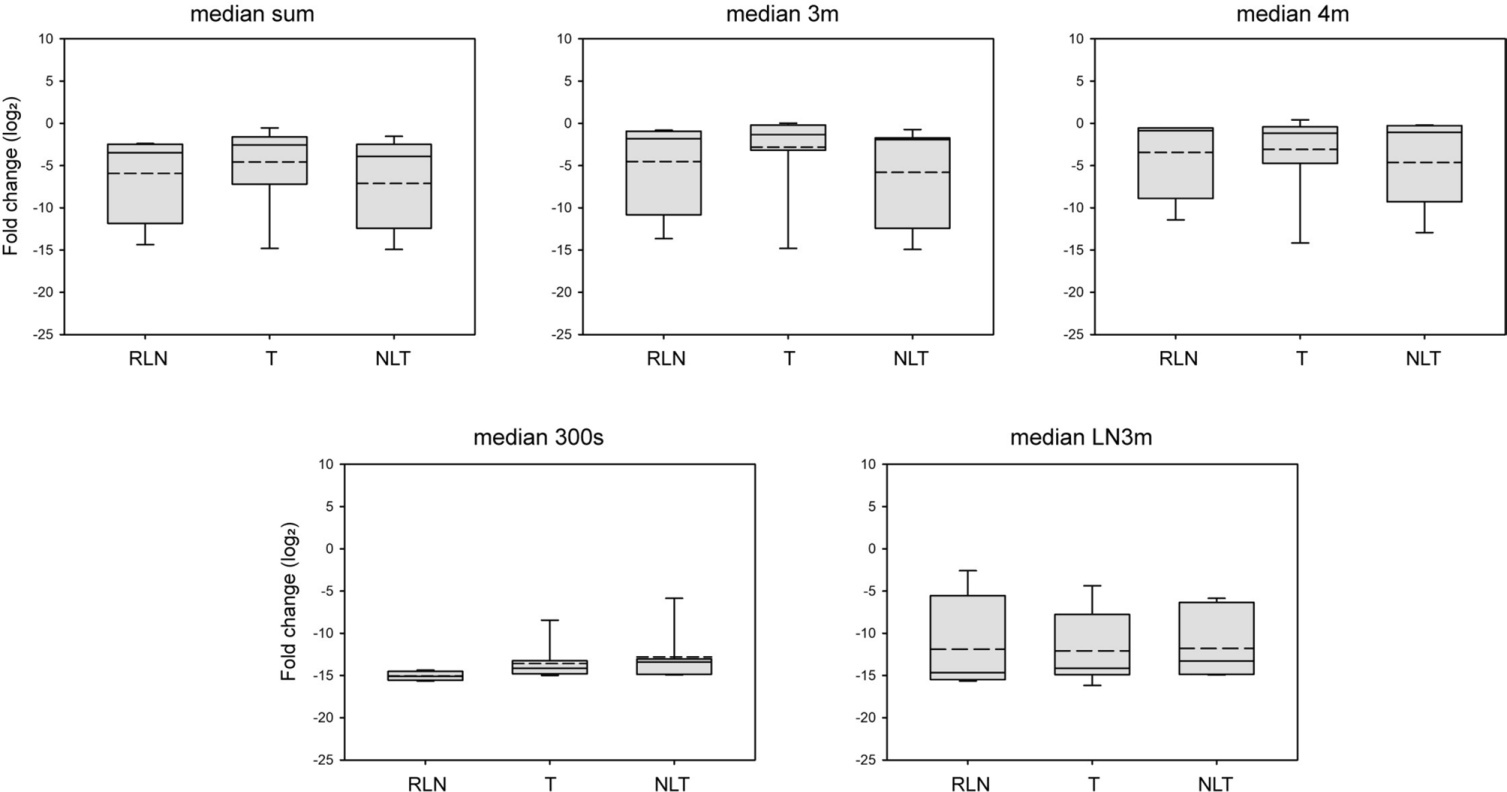
The serum levels of circulating miRNA indicators in distinct chemotherapy responders. The serum levels of the five previously defined indicators are represented in box plots. Solid lines represent means, and the medium dashed lines display the medians of datasets. Abbreviations stand for the following: RLN = reactive LNs, T: teratoma (metastatic LNs with living teratoma cells), and NLT: no living tumor (LNs with scar tissue or necrosis).

Our main goal was to facilitate the follow-up and monitoring of TCa patients' management with retroperitoneal LN metastases after chemotherapy. To assess the diagnostic ability of these pre-selected marker miRNAs, we re-analyzed the serum levels of the nine pre-selected marker miRNAs. The TCa sera samples were sorted into RLN, T, and NLT sub-groups according to the donors' histological ranks. Patients without LN tissue samples were excluded from these analyses. None of the miRNAs showed significant differences between the three-sample categories (Figure S2). The serum expression patterns of all the indicators defined by the previous examinations on sera and LN samples, median sum, 3 m, 4 m, 300 s, and LN3m, were also examined. In the case of median 300 s, all the indicators were unequally distributed and fluctuated throughout the samples (Fig. 5). The summarised sera expression pattern of miR-267, miR-371, and miR-373 had a narrow dispersion and displayed an increasing tendency in the T category compared to the RLN group; however, the difference was not significant. These data indicated that using the serum level patterns of circulating miRNAs individually or as combined indicators has substantial limitations in assessing metastatic TCa patients' therapeutic responses. To further refine and strengthen the diagnostic value of this methodology, more candidate miRNAs should be involved in the future.

## Conclusions and discussion

In this study, we successfully identified the presence of TGCT in blood sera with exceptional diagnostic accuracy. Early detection of TCa is crucial for improving patient survival^37^, and our research highlights the potential of miRNA expression as a highly sensitive and specific indicator for TCa compared to classical serum tumor markers (APF, β-hCG, and LDH). This breakthrough in diagnostic precision suggests that miRNA expression can play a pivotal role in future diagnostic evaluations and treatment response monitoring. Post-pubertal TGCT exhibits a low number of DNA mutations and somatic changes, with frequent observations of somatic mutations in the proto-oncogene *c-KIT*, encoding the receptor tyrosine kinase KIT, *KRAS* proto-oncogene, encoding the GTPase KRAS, and *NRAS* proto-oncogene, encoding the GTPase NRAS^38,39^. Additionally, *c-KIT* mutations^6^, pathogenic germline *CHEK2* variants^8^, and chromosomal alterations (gain of X, 7, 8, and 21 and loss of Y, 1p, 11, 13, and 18) are frequent^9^. The heritability of TGCT is estimated at 37–49%, with 78% susceptibility identified loci^40^. Major risk factors include a family history of TCa among first-degree relatives, the presence of a contralateral testicular tumor or germ cell in situ neoplasia, and testicular dysgenesis syndrome^41–43^. The standard diagnostic evaluation for localized TCa involves physical examination, ultrasound imaging, and applying classical serum tumor markers. For further assessment of metastatic status, treatment response monitoring, follow-up, contrast-enhanced CT or MR imaging of the abdominopelvic region is recommended^20^.

In the current landscape of cancer research, there is a growing emphasis on identifying novel and reliable biomarkers for tumor diagnostics. Among these, miRNAs have emerged as up-and-coming candidates. They can be quantified not only from solid tissues but also from circulating exosomes in the bloodstream, making them versatile and potentially invaluable for diagnostic purposes. MicroRNAs offer several advantages as biomarkers, including their detectability in the bloodstream, remarkable stability, and short half-life in comparison to classical markers used for TCa diganostics^25,30,44^. Our comprehensive analysis pinpointed six individual miRNAs (miR-19a, miR-21, miR-29a, miR-106b, miR-155, and miR-199a) with significant expression in post-chemotherapy TCa patients. Notably, the miR-21 + miR-29a + miR-106b cluster exhibited the highest sensitivity (96%) and specificity (78%) for detecting TCa. This finding underscores the cluster's potential as a valuable tool for both early detection and post-chemotherapy follow-up, pending further validation in subsequent research endeavors.

Furthermore, our study delved into tissue levels of the selected miRNAs in post-chemotherapy residual masses, revealing distinct expression patterns in RNL, NLT, and T groups. MiR-21, miR-155, and miR-373 were slightly elevated in teratoma compared to reactive LNs. Two miRNA clusters (miR-367 + miR-371a + miR-373 and miR-21 + miR-29a + miR-155) effectively differentiated between RLN and T groups although with relatively low specificity. Future studies should address the limitations of sample size and subgroup inequality, including viable GCT samples.

Despite the promising results obtained, it is crucial to acknowledge certain limitations in our study. The relatively low number of LN samples and the unequal sizes of subgroups may impact the generalizability of our findings. Future analyses should aim to include a more extensive and balanced sample set, particularly incorporating samples containing viable GCT to enhance the comprehensiveness of our research. Our observations underscore the intricate regulation of miRNA expression by different molecular mechanisms in blood serum and tumor tissue. It highlights the complexity of utilizing miRNAs as biomarkers and emphasizes the need for a nuanced interpretation of results across different biological matrices. In classifying TCa sera samples based on histological ranks (RNL, NLT, and T subgroups), circulating miRNA expression did not differ significantly among the three histological categories. It implies that utilizing serum-level patterns of circulating miRNAs may have limitations in assessing therapeutic responses in metastatic TCa patients. It becomes evident that our current panel of nine individual miRNAs and pre-established miRNA clusters may not fully capture the intricacies of the tumor microenvironment in serum. Future research endeavors should focus on identifying and studying additional candidate miRNAs to improve the diagnostic value and therapeutic response assessment in metastatic TCa patients.

### Supplementary Information

Supplementary Information.

## Data Availability

The data that support the findings of this study are available from [HCEMM] but restrictions apply to the availability of these data, which were used under license for the current study, and so are not publicly available. Data are however available from the authors upon reasonable request and with permission of [HCEMM]. The request should be sent to Tibor Pankotai (pankotai.tibor@szte.hu) as the corresponding author of the manuscript.
