# Supplementary material for: miR-21, miR-29a, and miR-106b: serum and tissue biomarkers with diagnostic potential in metastatic testicular cancer
Source: Sci Rep. 2024 Aug 30;14:20151. doi: 10.1038/s41598-024-70552-x (PMC11364861; doi:10.1038/s41598-024-70552-x)
Supplement: Supplementary file 1 — Supplementary Information. [file 41598_2024_70552_MOESM1_ESM.pdf]

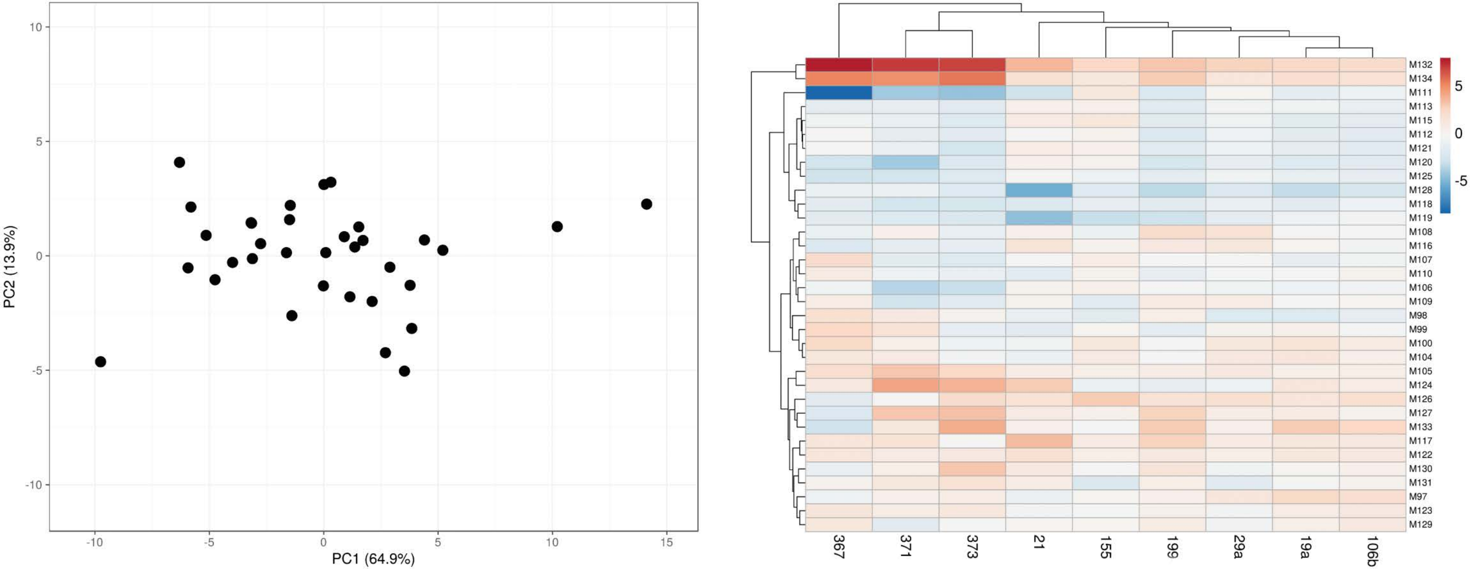

PC1 (64.9%)
PC2 (13.9%)
-10
-5
0
5
10
15
M132
M134
M111
M113
M115
M112
M121
M120
M125
M128
M118
M119
M108
M116
M107
M110
M106
M109
M98
M99
M100
M104
M105
M124
M126
M127
M133
M117
M122
M130
M131
M97
M123
M129
367
371
373
21
155
199
29a
19a
106b
5
0
-5

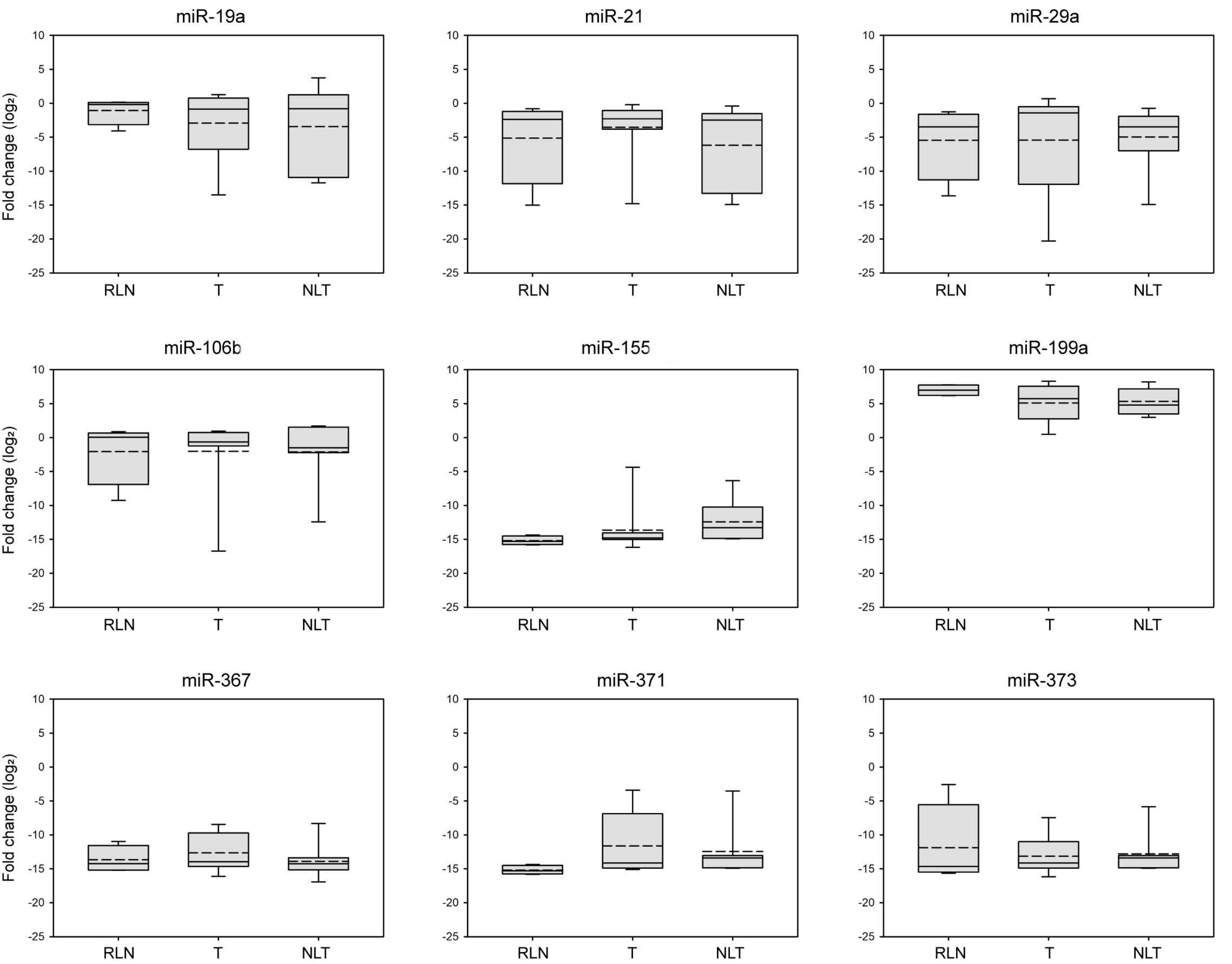
miR-19a
miR-21
miR-29a
miR-106b
miR-155
miR-199a
miR-367
miR-371
miR-373
Fold change (log₂)
10
5
0
-5
-10
-15
-20
-25
RLN
T
NLT

Supplementary Figure legends

Figure S1 Variance-based statistical evaluation of the expression of the nine oncomiRs in reactive LNs, teratoma-infiltrated metastatic LNs, and scar tissue/necrotic LNs removed from TCa patients after chemotherapy. No difference was observed in LN sample groups represented on PCA plot (A) and Heat map (B), which are generated with the ClustVis online tool. Abbreviations stand for PC = Principal Component, M and number combinations (right Y-axis of the heat map) = IDs of LN samples.

Figure S2 Medians of the normalized expressions of nine marker miRNAs were calculated and compared in reactive LNs, teratoma-infiltrated metastatic LNs, and necrotic LNs/scar tissues. The normalized tissue expression levels of miRNAs are indicated as log2 fold change. Abbreviations stand for the following: RLN = reactive LNs, T = teratoma (metastatic LNs with living teratoma cells), and NLT = no living tumor (LNs with scar tissue or necrosis).
